# Association between asthma and cardiovascular disease among a United States representative population

**DOI:** 10.1016/j.ijcrp.2025.200451

**Published:** 2025-06-06

**Authors:** Humza Naqvi, Charles D. Searles

**Affiliations:** a Alabama College of Osteopathic Medicine, Dothan, AL, USA; b Emory University School of Medicine, Atlanta, GA, USA; c Joseph Maxwell Cleland Atlanta VA Medical Center, Decatur, GA, USA

## 1 Introduction

Asthma is a common inflammatory condition mediated by proinflammatory cytokines and cells that have been shown to cause systemic inflammation and increase the risk of adverse outcomes for other systemic inflammatory conditions, such as coronary artery disease [1]. While the overall prevalence of asthma in the U.S. has remained relatively stable, environmental factors such as climate change and pollution have contributed to increases in asthma among different demographic groups, including military personnel who were exposed to the environmental hazards of Mideast war zones. For example, among Veterans who served in Iraq and Afghanistan, the prevalence of asthma tripled between 2003 and 2011 [2]. There are several postulated mechanisms for how asthma can increase the risk of coronary artery disease [3]. One pathophysiological mechanism is believed to be due to mast cell and eosinophil activation, which release inflammatory mediators that play a role in tissue remodeling, and ultimately atherosclerotic plaque formation [1]. However, there have been conflicting findings regarding the relationship between cardiovascular disease (CVD) and asthma among large international populations [3]. Our study provides further insight into this relationship by evaluating the association between asthma and coronary artery disease in a large, representative United States population.

## 2 Methods

We investigated the association between asthma and CVD among adults ages ≥20 years utilizing the National Health and Nutrition Examination Survey (NHANES) data from 2009 to 2014. NHANES is a survey conducted by the Centers for Disease Control and Prevention (CDC) that collects data from random individuals in the United States such as laboratory data, physical examinations, survey data, and demographic data. NHANES provides weighting variables to ensure that the data sets represent the demographics of the United States population. Outcome variables for CVD included a history of angina, myocardial infarction (MI), congestive heart failure (CHF), and stroke. In addition, severe asthma was defined as “having an emergency care visit for asthma in the past year.” Logistic regression analyses were conducted using Stata/BE 18.5. Subpopulation models were also constructed to evaluate the relationship between asthma and CVD by sex. Logistic regression models controlled for potential confounders, including for age, sex, race/ethnicity, tobacco smoking, diabetes, history of high blood pressure, history of high cholesterol, and BMI.

## 3 Results:

Approximately 14.7 % of individuals in our study reported being diagnosed with asthma. Of those individuals, 4.1 % reported a history of CHF, 4.2 % reported a history of angina, 4.8 % reported a history of MI, and 3.9 % reported a history of stroke (Table I). After adjusting for confounders (Table II legend), there was a significant association between asthma and history of angina (AOR 2.73, 95 % CI 2.06–3.62, p < 0.001), asthma and history of stroke (AOR 1.72, 95 % CI 1.30–2.27, p < 0.001), asthma and history of myocardial infarction (AOR 2.19, 95 % CI 1.58–3.03, p < 0.001), and asthma and history of congestive heart failure (AOR 1.96, 95 % CI 1.42–2.70, p < 0.001) (Table II).

**Table 1:** Demographics of subjects aged 20 years and older with and without asthma in NHANES 2009–2014.

Characteristic	Weighted % (95 % CI)^a^ Asthma^b^ (N = 2519)	Weighted % (95 % CI)^a^ No Asthma^b^ (N = 14,917)	P-value
CHF^c^			
Yes	4.1 (3.3–5.2)	2.1 (1.8–2.5)	**<0.0001**
No	95.9 (94.8–96.7)	97.9 (97.5–98.2)	
**Stroke**^d^			
Yes	3.9 (3.2–4.7)	2.6 (2.3–2.9)	**0.002**
No	96.1 (95.3–96.8)	97.4 (97.1–97.7)	
**Myocardial Infarction**^e^			
Yes	4.8 (3.9–6.0)	2.9 (2.6–3.3)	**<0.0001**
No	95.2 (94.0–96.1)	97.1 (96.7–97.4)	
**Angina**^f^			
Yes	4.2 (3.3–5.3)	1.7 (1.5–2.0)	**<0.0001**
No	95.9 (94.7–96.7)	98.3 (98.0–98.5)	
**Sex**			
Male	40.4 (37.9–42.9)	49.4 (48.5–50.2)	**<0.0001**
Female	59.6 (57.0–62.1)	50.6 (49.8–51.5)	
**Race/Ethnicity**			
Non-Hispanic White	69.5 (64.6–73.9)	66.3 (62.1–70.2)	**<0.0001**
Non-Hispanic Black	13.4 (10.9–16.5)	11.1 (9.4–13.1)	
Hispanic	10.8 (8.4–13.8)	14.74 (11.9–18.1)	
Other	6.3 (5.1–7.7)	7.9 (6.7–9.2)	
**Age**			
20–40 years	44.1 (41.5–46.6)	37.6 (35.6–39.6)	**<0.0001**
41–60 years	33.6 (31.7–35.6)	38.1 (36.7–39.5)	
61+ years	22.4 (20.3–24.6)	24.3 (23.1–25.6)	
**BMI**			
<18.5 kg/m^2^	1.3 (0.9–2.0)	1.7 (1.4–2.0)	**<0.0001**
18.5–24.9 kg/m^2^	25.2 (22.4–28.2)	29.0 (27.6–30.4)	
25–29.9 kg/m^2^	29.8 (27.7–32.0)	33.5 (32.2–34.9)	
30 kg/m^2^	43.7 (40.6–47.0)	35.8 (34.5–37.2)	

Values that are statistically significant (two-side P-value ≤0.05) are in bold.

BMI, Body Mass Index; CHF, Congestive Heart Failure; CI, Confidence Interval; NHANES, National Health and Nutrition Examination Survey.

a Weighted percentage was calculated using NHANES survey design parameters.

b Asthma status was assessed by the question “Has a doctor or other health professional ever told you that you have asthma?”

c Congestive heart failure status was assessed by the question “Has a doctor or other health professional ever told you that you had congestive heart failure?”

d Stroke status was assessed by the question “Has a doctor or other health professional ever told you that you had a stroke?”

e Myocardial infarction status was assessed by the question “Has a doctor or other health professional ever told you that you had a heart attack (also called myocardial infarction)?”

f Angina status was assessed by the question “Has a doctor or other health professional ever told you that you had angina, also called angina pectoris.

**Table 2:** Association between asthma and cardiovascular disease among US adults (ages ≥20 years) in NHANES 2009–2014.

**Variable**	OR (95 % CI)		P-value	
	**Unadjusted**	**Adjusted**^a^	**Unadjusted**	**Adjusted**^a^
*All participants*				
Angina	2.48 (1.91–3.23)	2.73 (2.06–3.62)	**<0.001**	**<0.001**
Stroke	1.51 (1.18–1.92)	1.72 (1.30–2.27)	**0.002**	**<0.001**
MI	1.69 (1.34–2.13)	2.19 (1.58–3.03)	**<0.001**	**<0.001**
CHF	1.97 (1.53–2.54)	1.96 (1.42–2.70)	**<0.001**	**<0.001**
*Males*				
Angina	2.19 (1.38–3.49)	2.92 (1.68–5.06)	**0.001**	**<0.001**
Stroke	0.86 (0.50–1.46)	1.32 (0.78–2.25)	0.6	0.3
MI	1.53 (1.00–2.33)	2.48 (1.38–4.45)	**0.05**	**0.003**
CHF	2.16 (1.65–2.83)	2.97 (2.03–4.36)	**<0.001**	**<0.001**
*Females*				
Angina	2.99 (1.92–4.66)	2.70 (1.61–4.51)	**<0.001**	**<0.001**
Stroke	1.94 (1.50–2.50)	1.94 (1.41–2.67)	**<0.001**	**<0.001**
MI	2.18 (1.60–2.98)	2.07 (1.40–3.05)	**<0.001**	**<0.001**
CHF	1.83 (1.20–2.80)	1.52 (0.88–2.62)	**0.006**	0.1
*Severe vs Mild asthma*				
Angina	2.82 (1.55–5.12)	2.74 (1.45–5.17)	**0.001**	**0.003**
Stroke	1.26 (0.68–2.33)	1.18 (0.52–2.66)	0.5	0.7
MI	1.64 (0.89–3.04)	2.34 (1.07–5.13)	0.1	**0.04**
CHF	1.61 (0.88–2.94)	2.28 (1.11–4.70)	0.1	**0.03**

Values that are statistically significant (two-side P-value ≤0.05) are in bold.

MI, Myocardial Infarction; BMI, Body Mass Index; CHF, Congestive Heart Failure; CI, Confidence Interval; NHANES, National Health and Nutrition Examination Survey; OR, Odds Ratio.

Severe asthma was defined as having an emergency care visit for asthma in the past year.

Mild asthma was defined as not having an emergency care visit for asthma in the past year.

∗ Weighted percentage was calculated using NHANES survey design parameters.

a Logistic regression models were adjusted for age, sex, race/ethnicity, tobacco smoking, diabetes, history of high blood pressure, history of high cholesterol, and BMI.

Subpopulation regression models revealed that among males there was no significant association between asthma and history of stroke (p = 0.3) (Table II). Additionally, among females there was no significant association between asthma and history of CHF (p = 0.1). Individuals with severe asthma were significantly more likely to report a history of CHF, MI, and angina compared to individuals with mild asthma (Table II). Moreover, individuals with severe asthma were more likely to report a history of CHF (AOR 2.28, 95 % CI 1.11–4.70, p = 0.03), MI (AOR 2.34, 95 % CI 1.07–5.13, p = 0.04), and angina (AOR 2.74, 95 % CI 1.45–5.17, p 0.003) compared to individuals with mild asthma (Table II).

## 4 Discussion

These findings reveal that after controlling for confounding risk factors, asthma has a significant association with self-reported history of angina, MI, CHF, and stroke. These findings indicate that the association between asthma and CVD may be independent of traditional CVD risk factors. Since patients with asthma have increased expression of inflammatory markers, the link between asthma and CVD may be due to underlying systemic inflammation present in asthma that leads production of prothrombotic factors [4]. Our results suggest that the association between asthma and CVD may differ by sex. Other studies have reported conflicting data on this issue. For example, it has been reported that females with asthma have a higher risk of CVD compared to males, while others have reported that males who had asthma were protected from coronary heart disease compared to males without asthma [3]. Moreover, other studies have reported that the asthma-associated risk of CVD was the same for men and women [3].

Our findings indicated that individuals with severe asthma were significantly more likely to report a history of CHF, MI, and angina compared to those with mild asthma. Currently, the impact of asthma severity on risk of CVD is uncertain. Choi et al. reported that more severe asthma increased the odds of having CVD compared to asthma alone [4]. In addition, individuals with severe work-related asthma were more likely to have CVD [5]. While our data supports an association between asthma and development of CVD, the study has several limitations, including self-reported data, the inability to control for impact of medications, and recall bias. Future studies utilizing International Classification of Diseases (ICD) codes in other large and diverse cohorts are needed to further verify the causal relationship between asthma and CVD.

## CRediT authorship contribution statement

**Humza Naqvi:** Data curation, Formal analysis, Writing – original draft. **Charles D. Searles:** Supervision, Writing – review & editing.

## Disclosures

None.

## Sources of funding

None.
